# New technologies to analyse protein function: an intrinsic disorder perspective

**DOI:** 10.12688/f1000research.20867.1

**Published:** 2020-02-10

**Authors:** Vladimir N. Uversky

**Affiliations:** 1 Department of Molecular Medicine and USF Health Byrd Alzheimer’s Research Institute, Morsani College of Medicine, University of South Florida, Tampa, FL, 33620, USA; 2 Laboratory of New Methods in Biology, Institute for Biological Instrumentation, Russian Academy of Sciences, Pushchino, Russian Federation

**Keywords:** intrinsically disordered protein, intrinsically disordered protein region, protein function, structure-function continuum

## Abstract

Functions of intrinsically disordered proteins do not require structure. Such structure-independent functionality has melted away the classic rigid “lock and key” representation of structure–function relationships in proteins, opening a new page in protein science, where molten keys operate on melted locks and where conformational flexibility and intrinsic disorder, structural plasticity and extreme malleability, multifunctionality and binding promiscuity represent a new-fangled reality. Analysis and understanding of this new reality require novel tools, and some of the techniques elaborated for the examination of intrinsically disordered protein functions are outlined in this review.

## Introduction to the disorder-based functionality: melted locks and molten keys

For more than a hundred years, the dominant model describing the molecular mechanism of protein functionality was the classic structure–function paradigm. This paradigm considered protein function in light of the “lock and key” hypothesis, where a unique biological function of a protein was considered to be the consequence of the presence of a unique and highly organized structure in its active site and where, in order to exert a chemical effect on each other, both a substrate and an enzyme have specific geometric shapes that fit exactly into each other, like a key specifically and uniquely fits to a lock ^1, 2^. In line with this hypothesis were numerous pieces of evidence generated by the crystal structures of proteins solved by x-ray diffraction, careful analysis of protein denaturation and unfolding, and many other observations, all indicating that specific functionality of a given protein is defined by a unique spatial positioning of its amino acid side chains and prosthetic groups, suggesting that such a specific spatial arrangement of functional groups in biologically active proteins is defined by their unique 3D structures predetermined by the unique amino acid sequences encoded in unique genes. These correlations were in line with the famous “one gene–one enzyme” hypothesis, where a gene encodes a single enzyme that affects a single step in a metabolic pathway ^3^. It is recognized now that the aforementioned “one gene–one enzyme” hypothesis is an oversimplification, and numerous observations fail to fit into or be explained by this model ^4^. Accumulated data challenged both the functional requirement of a unique structure in a biologically active protein and the absolute validity of the “one gene–one enzyme” conjecture, suggesting that the related paradigms should be changed ^5– 8^. In line with these considerations, it is recognized now that the complexity of biological systems is determined by protein diversification and not by the existence of a large number of distinct genes each encoding a unique protein ^9^. In fact, multiple means cause the dramatic and efficient increase in the size of a functional proteome in comparison with the size of a corresponding genome. These proteome-diversifying factors include the allelic variations (that is, single- or multiple-point mutations, insertions and deletions [indels], and single-nucleotide polymorphisms), different pre-translational mechanisms affecting genes (for example, production of numerous mRNA variants by the alternative splicing and mRNA editing), and changes induced in proteins by numerous post-translational modifications (PTMs) ^10– 14^. The result of this multilevel diversification that combines allelic variations, pre-translational alterations, and PTMs is the generation of multiple proteoforms, which are distinct protein molecules with different structures and diverse functions, from a single gene ^15^.

Furthermore, it is also recognized now that many protein functions do not require unique structure. These structure-less biologically active proteins carrying structure-independent functions are currently known as intrinsically disordered proteins (IDPs) or hybrid proteins containing ordered domains and IDP regions (IDPRs) ^5– 8, 16– 22^. These proteins, which were originally considered unique exceptions to the “lock and key” rule, are extremely common in nature; all proteomes of living organisms and viruses analysed so far possess noticeable levels of intrinsic disorder ^5, 19, 20, 22– 41^; and the penetrance of disorder increases with the increase in the organism complexity ^19, 23– 25, 42^. As an example, the fraction of proteins predicted to have long IDPRs (that is, disordered regions exceeding 30 consecutive residues) increases from Bacteria and Archaea to Eukaryota ^23, 24, 26, 28, 43^. The increased amount of disorder in eukaryotes is attributed to the increased roles of their cellular signalling that often relies on IDPs/IDPRs ^5, 6, 8, 18, 44– 47^. Also, just a small fraction of proteins with known crystal structures in the Protein Data Bank are entirely devoid of disorder ^48, 49^. An important feature of IDPs/IDPRs is their exceptional spatiotemporal heterogeneity, where different regions of a given protein can be ordered (or disordered) to a different degree ^50, 51^. Therefore, the overall structure of functional proteins represents a continuous spectrum of conformations with a different degree and depth of disorder ^50^, thereby generating a complex protein structural space that defines a structure-disorder continuum with no clear boundary between ordered and disordered proteins/regions ^50^. The presence of the aforementioned different levels and depths of intrinsic disorder delineates the mosaic structure of proteins, which typically contain foldons (that is, independently foldable regions), inducible foldons (disordered regions that can fold at interaction with a binding partner), morphing inducible foldons (disordered regions that can fold differently at interaction with a different binding partner), semi-foldons (IDPRs that are always in the semi-folded state), non-foldons (IDPRs with entropic chain activities), and unfoldons (or conditionally disordered protein regions, which, in order to become functional or to make a protein active, have to undergo order-to-disorder transition) ^50^. Obviously, the presence of intrinsic disorder and conformational flexibility in proteins contributes to their structural and functional heterogeneity, representing additional means for generating proteoforms ^52^. In fact, since any protein exists as a dynamic conformational ensemble, members of which have different structures (their structural differences could be rather subtle, as in the case of ordered proteins, or rather substantial, as in the case of IDPs/IDPRs) and potentially different functions, it can be considered a basic (or intrinsic or conformational) proteoform. Such a conformational proteoform is different from the inducible proteoform that originates from the various alterations (PTMs, mutations, or consequences of alternative splicing) of the canonical protein sequence and that represents a mixture of these various forms. Obviously, since it also represents a structural ensemble, any member of the inducible or modified proteoform (that is, any mutated, modified, or alternatively spliced form) is itself a conformational proteoform ^52^. Finally, since protein function, interaction with specific partners, or placement inside the natural cellular environment can also affect the structural ensemble of both basic and induced proteoforms, functionality per se can be considered a factor generating new functioning proteoforms. As a result, instead of being depicted as an oversimplified “one gene–one protein” view, the actual gene–protein relationship is much more complex, being described by the “one gene–many proteins–many functions” model ^52, 53^. Therefore, a correlation between protein structure and function represents a “protein structure–function continuum”, where at any given moment, any given protein exists as a dynamic conformational ensemble containing multiple proteoforms (conformational/basic, inducible/modified, and functioning) characterized by diverse structural features and various functions ^52^.

Concluding this section, we need to emphasize that the presence of intrinsic disorder and conformational flexibility in proteins changed the rigid “lock and key” model proposed for the description of the general molecular mechanisms of protein function. Although “lock and key” (or its modification in a form of induced fit) can be used for the description of catalytic activities of some enzymes, many other protein functions (for example, recognition, regulation, signalling, and promiscuous binding) do not fit into this rigid view since, owing to the presence of disorder and flexibility, the locks are melted and the keys are molten. This also suggests that some novel approaches are needed to analyse intrinsic disorder-based functionality. The goal of this article is to shed some light on this problem by presenting the most recent advances in the analysis of protein disorder-based functionality.

## Looking at the disorder-based functionality of proteins

### Laboratory techniques for the analysis of protein–protein interactions

Traditional analysis of protein functionality was mostly centred on the development of means for accurate characterization of enzymatic activity or ligand binding (or both) of a protein *in vitro* and *in vivo* and development of related molecular mechanisms. Although enzymatic catalysis is not among the disorder-based protein functions, some of the techniques elaborated for the analysis of the interactivity of ordered proteins can be successfully used for the functional characterization of IDPs. Biophysical techniques that are typically used to study protein–partner interactions are designed either to investigate thermodynamics or kinetics (or both) of the binding or to characterize the structural changes associated with the interactions. Many of these techniques are suitable for the analysis of both order-based and disorder-based protein interactions although the IDP-centred interactions involve a variety of binding modes, ranging from the folding upon binding mechanism to the formation of dynamic fuzzy complexes. Thermodynamic-focused techniques for the analysis of protein–partner interactions include isothermal titration calorimetry ^54, 55^ and surface plasmon resonance (SPR) ^56^, whereas dissociation constants can be measured by dynamic light scattering ^57^ and analytical ultracentrifugation ^58^. All of these techniques can determine dissociation constants. In addition, SPR can determine k _on_ and k _off_ of binding events ^56^. Although, traditionally, the major technique for the analysis of binding-induced structural changes in proteins was x-ray crystallography, this tool provides a static 3D picture of a protein complex and therefore has rather limited application to IDPs/IDPRs (with the obvious exception of the cases when disordered protein or region folds at interaction with the specific partner). Among other experimental techniques for the analysis of binding-induced structural changes are small-angle x-ray scattering (SAXS) ^59, 60^, single-molecule Förster resonance energy transfer (smFRET) (that analyses protein conformations without ensemble averaging and kinetics without interference from asynchronous processes) ^61– 65^, electron paramagnetic resonance (EPR) ^64, 66, 67^, and hydrogen/deuterium exchange (HDX) mass spectrometry ^68– 71^. Although IDPs/IDPRs are commonly involved in transient protein–protein interactions (that is, interactions characterized by the K _D_ values in the micromolar to millimolar range), which are crucial for cell signalling, characterization of such interactions at the atomic-resolution level is rather challenging by the majority of conventional techniques. However, such interactions can be analysed by using solution nuclear magnetic resonance (NMR) spectroscopy ^72– 76^, including diamagnetic and paramagnetic (for example, paramagnetic relaxation enhancement) techniques ^77^. Peculiarities of the application of NMR for the analysis of IDPs/IDPRs and disorder-based protein complexes are detailed in several recent reviews ^72, 75^. Importantly, smFRET ^78, 79^ and NMR ^80– 82^ can be successfully used for the in-cell analysis of IDPs and their interactions. It was also pointed out that the most appropriate and eloquent description of the structure and dynamics of IDPs and IDP-based complexes could be achieved via the combined use of several aforementioned techniques, such as NMR, smFRET, and SAXS enhanced by the molecular dynamic simulations, since complementary experimental data from these techniques ensure important and meaningful constraints for computational simulations ^83, 84^. In line with these developments, several groups are developing new approaches for the computational descriptions of disordered ensembles ^85– 99^. Furthermore, an openly accessible database of structural ensembles of intrinsically disordered and unfolded proteins, pE-DB ( http://pedb.vib.be), was created to promote the elaboration of novel modelling approaches and to allow a better understanding of disorder-based functionality ^100, 101^.

Illustrating the remarkable power of NMR spectroscopy when applied to the functional and structural analysis of disorder-based interactions, a recent study provided a structural characterization of an intriguing complex formed between two IDPs: human histone H1 and its nuclear chaperone prothymosin-alpha ^102^. Although these proteins formed a highly specific complex with picomolar affinity, they completely retained their highly disordered nature, long-range flexibility, and overall highly dynamic character ^102^. This complex is an extreme case of an IDP-driven polyelectrostatic binding mechanism proposed as a result of the NMR-based analysis of a complex between the polyvalent intrinsically disordered cyclin-dependent kinase inhibitor Sic1 and its ordered partner, SCF ubiquitin ligase subunit Cdc4 ^103^. This Sic1–Cdc4 complex is held together by cumulative electrostatic interactions between the numerous phosphorylated sites of Sic1 and a single binding site of Cdc4; the binding strength is dependent on the phosphorylation degree of Sic1, and Sic1 remains largely disordered in its Cdc4-bound state ^103^.

Multivalent interactions between IDPs that are not accompanied by noticeable structural changes are directly linked to the biogenesis of the proteinaceous membrane-less organelles (PMLOs), which are abundant in cytoplasm, nucleus, and mitochondria of various cells and which play a number of important roles in the organization of various intracellular processes ^104, 105^. PMLOs are related to various biological processes compartmentalized in diverse regions of the cell ^106^, are able to facilitate and respond to various biological functions and stimuli ^107^, and therefore are considered important players in cellular life. PMLOs are highly dynamic but stable, protein-only or protein–nucleic acid assemblages characterized by cell size–dependent dimensions ^108^, whose structural integrity and biogenesis are exclusively determined by protein–protein, protein–RNA, or protein–DNA interactions or a combination of these ^109, 110^. These liquid droplets are formed via the intracellular liquid–liquid phase transitions (LLPTs) or the intracellular liquid–liquid demixing phase separation ^108, 111^ initiated by the colocalization of molecules at high concentrations within a small cellular micro-domain ^112, 113^. Biogenesis of PMLOs is a highly controllable and reversible process, and formation of PMLOs is triggered by changes in the concentrations of proteins undergoing LLPT, changes in the concentrations of specific small molecules or salts, changes in osmolarity, and changes in the pH or temperature (or both) of the solution or by various PTMs and alternative splicing of the phase-forming proteins, by the binding of these proteins to some definite partners, or by changes in other environmental conditions that affect the protein–protein or protein–nucleic acid interactions ^108, 111, 114– 116^. PMLOs are very large (detectable by light microscope), liquid-like assemblages which are not covered by the membranes and whose components are involved in direct contact and exchange with the PMLO environment ^112, 113^. As a result, PMLOs are characterized by liquid-like behaviour, being capable of wetting, dripping, and forming spherical structures upon fusion ^108, 117– 119^. Since proteins driving LLPTs are intrinsically disordered or contain IDPRs ^120^, PMLOs represent an intricate form of the disorder-based protein complexes ^104, 105, 121^, which are commonly formed without noticeable structural changes in the proteins undergoing LLPTs ^122^. This conclusion is supported by the NMR analysis of several PMLOs or liquid droplets such as in the case of the Alzheimer-related protein tau ^123, 124^, elastin-like polypeptides (ELPs) ^125^, the low-complexity domain of the RNA-binding protein fused in sarcoma (FUS) ^126^, heterogeneous nuclear ribonucleoprotein A2 (hnRNPA2) ^127^, and the intrinsically disordered N-terminal 236 residues of the germ-granule protein Ddx4 ^128^. Techniques that can be used for the analysis of the dynamics, structure, morphology, and rheology of phase-separated droplets and PMLOs and their components *in vitro* and in live cells were systematically analysed in a recent review ^129^. Special emphasis was put on the suitability of single-molecule fluorescence methods for the characterization of functional dynamics of PMLOs ^130^, on the use of fluorescence recovery after photobleaching (FRAP) as a technique of first choice for assessing fluidity of PMLOs and phase-separated droplets and to estimate protein diffusion coefficients ^131^, and dual-colour fluorescence cross-correlation spectroscopy (FCCS) for the analysis of concentrations, diffusion characteristics and interactions of two fluorescent species in solution ^132^.

The liquid-like nature of PMLOs and phase-separated droplets can affect and modulate functions of their constituents, which are accumulated within droplets at high concentrations but remain dynamic. In line with this hypothesis, the low-density structure of PMLOs in the *Xenopus* oocyte nucleus was shown to determine the access to the macromolecules within these PMLOs from the nucleoplasm ^133^. PMLOs can also act as liquid-phase micro-reactors, where the cytoplasmic reactions are accelerated because of the increased concentrations of related RNA and protein components ^108, 134, 135^. PMLOs can also serve as a means for recruitment and concentration of specific proteins, as seen in Negri bodies (NBs), which are cytoplasmic liquid organelles in which viral RNAs (mRNAs as well as genomic and antigenomic RNAs) are synthesized ^136^. Neuronal ribonucleoprotein (RNP) particles, or granules that concentrate specific sets of mRNAs and regulatory proteins, serve as dynamic sensors of localized signals and play a dual role in the translation of associated mRNAs, preventing mRNA translation at rest and ensuring local protein synthesis upon activation ^137^.

LLPTs and PMLOs are illustrative examples of the disorder-based emergent behaviour of proteins ^50, 138– 140^. Another example of the emergent behaviour is given by the spatiotemporal oscillations of the Min protein system (MinD, MinC and MinE) that moves from pole to pole of the rod-shaped *Escherichia coli* cells with the intrinsic wavelength comparable to the size of the *E. coli* cell ^141^. Oscillating movements of this system are required for the spatial regulation of the positioning of the cytokinetic Z ring that determines the division plane ^142– 144^. Such oscillations can be visualized if the components of this system are fluorescently labelled ^145– 147^. Furthermore, on the supported lipid bilayers *in vitro*, these Min proteins self-organize to form traveling protein surface waves emerging from the repetitive binding-detaching cycles of proteins to the membrane ^143, 144, 148, 149^. Also, depending on the peculiarities of their environment, MinD and MinE were shown to self-organize into a wide variety of patterns ^150^.

Bioimaging is a commonly used technique for the quantification of intracellular protein–protein interactions (PPIs). Here, the presence of molecular interactions is judged by the analysis of spatial colocalization between the different populations of differently labelled molecules in the field of view (FOV) of dual- or multiple-channel fluorescence microscope ^151^. Colocalization is evaluated by pixel-based methods or object-based methods ^151^. In the first case, the image generated by the fluorescence microscope is analysed to measure global correlation coefficients between pixel intensities in different colour channels that allow finding and quantification of overlapping pixel intensities in different channels ^152^. In the second case, the objects (molecules) are first segmented and then represented as points through coordinates of their mass centre in the delimited FOV and then their spatial distributions are analysed ^153, 154^. A systematic study published in 2015 compared pixel-based and object-based methods for finding colocalization in synthetic and biological images and revealed that data generated by the object-based methods are more statistically robust than the results of pixel-based approaches ^151^.

PPIs *in vitro* and *in vivo* are traditionally analysed by using the affinity purification-based pull-down assays ^155^ or co-immunoprecipitation (coIP) experiments ^156^ allowing the direct detection of physical interactions. Here, either purified and tagged protein is used as a “bait” to bind any interacting proteins (pull-down assays) or antibody against a target protein is used to immunoprecipitate the complexes containing the target protein (coIP). Although CoIP and pull-down assays are typically used as “yes-no” tools for showing the presence or absence of PPIs, it was recently shown that the dissociation constant (K _D_) of complexes formed by two purified proteins can be measured by using the quantitative pull-down assay ^157^. However, these two techniques are typically limited to the high-affinity binding and therefore are not easily transferable to the analysis of disorder-based interactions, which are often weak. This caveat can be overcome by using chemical ^158^ or photo-affinity ^159^ cross-linking of samples before conducting pull-down and CoIP assays. Chemical and photo-affinity cross-linking combined with mass spectrometry (XL-MS) is another technique for the analysis of weak and transient PPIs ^159– 164^. The use of genetically encoded photo-crosslinkers using natural amino acid analogues that contain a photo-affinity group as the warhead and that can be site-specifically incorporated into a protein of interest to covalently trap non-covalent PPIs under living conditions represents a promising development in this area ^165^.

One of the commonly used approaches for investigating PPIs in living systems is a genetic approach: yeast-two-hybrid (YTH) screening ^166– 168^. Here, interaction between two proteins, called bait and prey, activates reporter genes that enable yeast growth on specific media or a colour reaction ^168^. In 2015, high-affinity binders to transiently structured IDP, the prokaryotic ubiquitin-like protein Pup, and its unstructured segments were identified and characterized at atomic resolution by using the YTH-selected peptide aptamers and in-cell NMR ^169^. Similarly, a combination of YTH screenings with NMR spectroscopy, cross-linking experiments, and competition-binding assays was recently used to characterize the interactivity of a long IDPR linking the KIX domain (kinase-inducible domain [KID] interacting domain) and bromodomain of CBP (cAMP response element-binding [CREB]-binding protein) termed ID3 and to show that ID3 binds to the intrinsically disordered RNA-binding Zinc-finger protein 106 (ZFP106), and both interactors maintained disorder in their bound states ^170^. Recently, YTH assay was used to compare mutational robustness of the intrinsically disordered viral protein VPg and of its interactor eIF4E using libraries of mutant forms of both VPg and eIF4E ^171^. This study revealed that VPg was significantly more robust against mutations than eIF4E ^171^.

Another tool for the analysis of weak PPIs is the bimolecular fluorescence complementation (BiFC) assay, which uses the ability of two non-fluorescent fragments of a fluorescent protein to associate and form a fluorescent complex, and association is facilitated when they are fused to two interacting proteins ^172, 173^. BiFC was successfully used for the *in planta* analysis of homo- and hetero-dimerization of the intrinsically disordered dehydrins from *Arabidopsis thaliana*, AtCOR47, AtERD10 and AtRAB18 ^174^, and for the analysis of interactivity of another Arabidopsis protein, histone deacetylase complex 1 (HDC1) protein ^175^.

Finally, among other experimental tools used for the analysis of PPIs are various proximity-dependent labelling (PDL) approaches, where the target protein has to be fused with an enzyme capable of catalytic attachment of a reactive molecule to the interacting partners in a distance-dependent manner (typically a few tens to hundreds of nanometers) ^176– 178^. One of these PDL systems is a proximity-dependent biotin identification (BioID) approach that uses biotin ligase BirA as an enzyme catalysing the biotinylation of target protein in the presence of biotin and that uses subsequent streptavidin-mediated pull-down and mass spectrometry analysis for the identification of interacting proteins ^179, 180^. Recently, it was shown that biotinylation-based proximity labelling is biased by structural features of target proteins, causing enrichment of cellular biotinylation events within the IDPRs of protein targets ^181^. In addition to biotin ligase, proximity labelling can be conducted by some peroxidase enzymes, which, in the presence hydrogen peroxide, can generate short-lived free radicals (for example, from phenolic compounds) that represent the enzyme-generated reagents that can covalently label neighbouring proteins ^178, 182^.

### Computational approaches for the analysis of disorder-based functionality

Among the important features of IDPs/IDPRs associated with their functionality are the ability to undergo at least partial folding at interaction with specific partners ^5, 8, 18, 44– 47, 183– 189^ and the capability to bind to multiple partners and gain very different structures in the bound state ^190– 196^, which increases complexity of the disorder-based interactomes ^197^. Often, such foldable IDPRs are engaged in recognition function of IDPs and therefore are known as molecular recognition features ^188, 198– 201^. Since such molecular recognition features (MoRFs) (for example, sub-regions of IDPs/IDPRs capable of binding-induced folding) are characterized by specific features (they cannot fold by themselves but have the potential to do so when a specific partner is present), they can be rather accurately predicted from the protein amino acid sequence ^202^. There are numerous computational tools for finding disorder-based interactions sites in proteins, which are grouped into three major classes: tools looking for MoRFs (alpha-MoRFpred ^188, 200^, MoRFpred ^203^, MFSPSSMpred ^204^, MoRFChiBi ^205, 206^, fMoRFpred ^207^, retro-MoRF ^208^, and DISOPRED3 ^209^); algorithms such as PepBindPred ^210^, ANCHOR ^211, 212^ and disoRDPbind ^213^ to find generic disordered protein-binding regions; and a method for finding short linear sequence motifs (SLiMs), SLiMpred ^214^. Although all of these tools analyse the capability of a target protein to be engaged in PPIs, disoRDPbind also predicts the protein region capable of binding to DNA and RNA ^213^. There is also a tool for finding disordered flexible linker regions that serve as linkers/spacers in multi-domain proteins or between structured constituents in protein domains: the DFLpred method ^215^. Peculiarities, advantages and disadvantages of all of these techniques, together with the 32 tools for the prediction of intrinsic disorder predisposition of a query protein, were carefully analysed and compared in a recent comprehensive review ^202^. Recently, Zarin *et al*. did a comprehensive evolutionary computational analysis to search for molecular features that are preserved in the amino acid sequences of orthologous IDPRs ^216^. This analysis revealed that orthologous IDPRs frequently contain multiple “evolutionary signatures” (that is, molecular features, which are preserved within these IDPRs and are associated with multiple functional annotations and phenotypes). Based on these observations, it was suggested that such evolutionary signatures could be used for the prediction of functionality of IDPRs from their amino acid sequences ^216^.

Another important feature of disorder-based functions is their regulation by numerous PTMs ^5, 6, 44, 45, 217, 218^. Therefore, prediction of localization of PTM sites within the amino acid sequences of IDPs and IDPRs represents an important direction in computational analysis of disorder-based functionality. In fact, systematic bioinformatic analyses of the peculiarities of the IDP/IDPR-located display sites targeted for PTMs and their adjacent regions demonstrated that their sequence attributes (such as amino acid compositions and sequence complexity, hydrophobicity, and charge) are rather similar to those of IDPRs. These observations define the potential predictability of such disorder-centred PTM sites and were used for the development of disorder-focused predictors of protein phosphorylation ^217^, methylation ^219^, ubiquitination ^220^, and S-palmitoylation ^221^, a unified sequence-based predictor of 23 types of PTM sites, which can be used for finding protein regions that undergo multiple homologous or heterologous PTM events and for finding shared PTM sites (that is, sites modified by more than one type of PTM) ^218^.

Disorder status and potential disorder-related information for a query protein can be retrieved from the D ^2^P ^2^ database ( http://d2p2.pro/) ^222^, which is a resource of pre-computed disorder predictions for a large library of proteins from completely sequenced genomes ^222^. In a visually attractive form, D ^2^P ^2^ generates a functional disorder profile of a query protein that includes outputs of nine per-residue disorder predictors, represents positions of functional domains, shows a gradient bar reflecting the consensus of nine disorder predictors, where the increase in strength of correlation is shown by colour change from white to dark green, and also indicates location of the predicted disorder-based binding sites (MoRFs) and positions of various PTMs ^222^.

Finally, localization of various functional short linear motifs, SLiMs, in a query protein can be assessed by the eukaryotic linear motif (ELM) resource ( http://elm.eu.org/), which is a collection of manually annotated SLiM instances curated from experimental literature ^223, 224^. SLiMs are composed of short stretches of adjacent amino acids and can be found in IDPRs of many proteins. They are short, compact, degenerate peptide segments that act as protein interaction sites and are essential for almost all cellular processes ^223^. An ELM resource can also be used for finding potential SLiMs in a query protein. It filters out globular domains and retains predicted SLiMs associated with various functions ^223, 224^. There are six types of annotations for the SLiMs that are described by the ELM server ^223, 224^: motifs for targeting to subcellular compartments (ELM_TRG), degron motifs that play a role in polyubiquitylation and targeting of proteins to proteasomal degradation (ELM_DEG), motifs that act as proteolytic cleavage sites (ELM_CLV), ligand binding motifs (ELM_LIG), docking motifs (ELM_DOC), and sites for PTMs (ELM_MOD) ^223, 224^.

One more important recent direction in the elaboration of computational tools for functional analysis of IDPs and IDPRs is related to the development of methods for prediction of liquid–liquid phase separation (LLPS) and finding phase-separating proteins (PSPs). In fact, although the analysis of LLPTs and PMLOs is a rapidly developing field that clearly attracts significant attention of multiple researchers, general knowledge of the prevalence and distribution of PSPs is still rather rudimentary. Therefore, tools for LLPS and PSP predictions are in high demand. Recently, information on the first-generation PSP predictors and their basic principles was summarized by Vernon *et al*. ^225^. Among these first-generation PSP predictors are the following:

- Prion-like amino acid composition (PLAAC) tool for finding PSPs ^226^ on the basis of identifying prion-like domains ^227^;
- A tool for finding PSPs on the basis of the similarity of sequence composition and residue spacing (statistical map of FG and RG) to fingerprints of PMLO-forming features of a disordered nuage protein Ddx4 ^122^;
- PScore that evaluates the expected number of long-range π–π interactions involving non-aromatic groups in a query protein ^228^;
- LARKS tool for finding, in query proteins, low-complexity aromatic-rich kinked segments that are potentially related to the formation of subcellular membrane-less assemblies ^229^;
- R+Y model for the evaluation of the content in a query protein of arginine and tyrosine residues that can be engaged in cation–π interactions serving as drivers of phase separation ^230^;
- the *cat*GRANULE algorithm that predicts PCPs by evaluating intrinsic disorder and nucleic acid binding propensities; sequence length; and arginine, glycine and phenylalanine content (R, G, F), which are known to be enriched in some PCPs ^231^;
- PSPer uses the hidden Markov model for prediction of PSPs and considers the presence in a query protein of prion-like domains, IDPRs, arginine-rich domains, RNA recognition motifs, and other features ^232^;
- PSPredictor, which is a machine learning tool for sequence-based prediction of PSPs ^233^.

Another illustration of the interest of researchers in LLPS and PMLOs is the recent development of an LLPSDB database ( http://bio-comp.org.cn/llpsdb) that provides comprehensive information on proteins undergoing LLPS *in vitro* and contains 1182 entries describing 273 independent proteins and 2394 specific conditions ^234^.

## Concluding remarks

Although IDPs/IDPRs were largely ignored for most of the existence of protein science, it is now clear that IDPs and disorder-based functions represent a new reality. Originally, the field of un-structural biology stood up as an attempt to explain many cases of rare exceptions (that is, proteins that fall outside of the classic structure–function paradigm with its “rigid” view of protein functionality as “lock and key” or “induced fit” models). However, in light of the broad acceptance of the new un-structural biology paradigm, one should keep in mind that it would be a clear mistake to continue contradistinguishing and opposing ordered proteins and IDPs, as they work together in a living cell, indicating that understanding and explanation of the protein dynamics and functionality require a tandem action of the disciplines of structural and un-structural biology ^235^. In fact, since different disorder-centred functions complement (mostly catalytic) activities of ordered proteins, structure and disorder represent a unity of opposites or *coincidentia oppositorum*. On the other hand, an actual line between order and disorder is elusive and structural and un-structural biology should not be opposed but united since they clearly complement one other ^235^. Therefore, a complete understanding of the biological functionality at the proteome level requires careful consideration of both order- and disorder-based protein functions and only such a united approach can ensure the previously unattainable comprehension of biological complexity. On the other hand, structural and functional characterization of ordered and disordered proteins requires very different methodological approaches, and an analysis of hybrid proteins remains a challenging task. In fact, as was pointed out, the current literature is focused mostly on fully ordered or fully disordered proteins, generating an immense “grey” area, where order and disorder are mixed and resulting in an incomplete understanding of the diverse mechanisms and functions used by hybrid proteins ^235^.

## Abbreviations

BiFC, bimolecular fluorescence complementation; coIP, co-immunoprecipitation; ELM, eukaryotic linear motif; FOV, field of view; ID3, IDPR linking the KIX domain and bromodomain of CBP; IDP, intrinsically disordered protein; IDPR, intrinsically disordered protein region; LLPS, liquid–liquid phase separation; LLPT, liquid–liquid phase transition; MoRF, molecular recognition feature; NMR, nuclear magnetic resonance; PDL, proximity-dependent labelling; PMLO, proteinaceous membrane-less organelle; PPI, protein–protein interaction; PSP, phase-separating protein; PTM, post-translational modification; SAXS, small-angle x-ray scattering; SLiM, short linear sequence motif; smFRET single-molecule Förster resonance energy transfer; SPR, surface plasmon resonance; YTH, yeast-two-hybrid
